# Adjustment for treatment changes in epilepsy trials: A comparison of causal methods for time-to-event outcomes

**DOI:** 10.1177/0962280217735560

**Published:** 2017-11-08

**Authors:** Susanna Dodd, Paula Williamson, Ian R White

**Affiliations:** 1 MRC North West Hub for Trials Methodology Research, Department of Biostatistics, Institute of Translational Medicine, University of Liverpool, Liverpool, UK; 2 MRC Biostatistics Unit, Cambridge Institute of Public Health, Cambridge, UK; 3 MRC Clinical Trials Unit at UCL, Institute of Clinical Trials & Methodology, London, UK

**Keywords:** Non-adherence, non-compliance, departure from randomised treatment, trial analysis, causal effect modelling

## Abstract

**Background:**

When trials are subject to departures from randomised treatment, simple statistical methods that aim to estimate treatment efficacy, such as per protocol or as treated analyses, typically introduce selection bias. More appropriate methods to adjust for departure from randomised treatment are rarely employed, primarily due to their complexity and unfamiliarity. We demonstrate the use of causal methodologies for the production of estimands with valid causal interpretation for time-to-event outcomes in the analysis of a complex epilepsy trial, as an example to guide non-specialist analysts undertaking similar analyses.

**Methods:**

Two causal methods, the structural failure time model and inverse probability of censoring weighting, are adapted to allow for skewed time-varying confounders, competing reasons for treatment changes and a complicated time to remission outcome. We demonstrate the impact of various factors: choice of method (structural failure time model versus inverse probability of censoring weighting), model for inverse probability of censoring weighting (pooled logistic regression versus Cox models), time interval (for creating panel data for time-varying confounders and outcome), choice of confounders and (in pooled logistic regression) use of splines to estimate underlying risk.

**Results:**

The structural failure time model could adjust for switches between trial treatments but had limited ability to adjust for the other treatment changes that occurred in this epilepsy trial. Inverse probability of censoring weighting was able to adjust for all treatment changes and demonstrated very similar results with Cox and pooled logistic regression models. Accounting for increasing numbers of time-varying confounders and reasons for treatment change suggested a more pronounced advantage of the control treatment than that obtained using intention to treat.

**Conclusions:**

In a complex trial featuring a remission outcome, underlying assumptions of the structural failure time model are likely to be violated, and inverse probability of censoring weighting may provide the most useful option, assuming availability of appropriate data and sufficient sample sizes. Recommendations are provided for analysts when considering which of these methods should be applied in a given trial setting.

## 1 Background

### 1.1 Adjusting for departure from randomised treatment

Departure from randomised treatment is common in trials with survival outcomes.^1^ In the presence of such departures, analysis according to randomisation (known as intention to treat (ITT) analysis) estimates effectiveness of treatment policy, as opposed to treatment efficacy. When interest lies in estimating treatment efficacy, trials are often analysed using simple methods such as ‘per protocol’ (PP) and ‘as treated’ (AT) analyses, which typically introduce selection or confounding bias by analysing according to treatment received.^2^ This bias occurs because departure from randomised treatment is often clinically indicated, resulting in systematic differences between patients who do and do not adhere to their assigned intervention. For example, patients who are not experiencing benefit from treatment are more likely to switch to alternative treatments; patients who experience adverse effects may decide to withdraw from treatment; or patients who are motivated to persevere with their assigned treatment, despite adverse effects or other challenges, may also be likely to adhere with other recommendations, such as lifestyle changes, which in turn impact on their prognosis. Therefore, compliance with assigned treatment is often associated with outcome and cannot replace randomisation in statistical analysis as an unconfounded predictor of outcome.

More appropriate causal methods, which seek to prevent such bias when estimating treatment efficacy, are available but rarely implemented.^1^ Two particular methods which are appropriate for estimating causal effects on survival outcomes, namely the structural failure time model (SFTM) and inverse probability of censoring weighting (IPCW) method, were described by Watkins et al.^3^ in the context of oncology trials featuring switching from control to experimental treatment only (typically on disease progression).

This underuse of appropriate causal methods may be due to their relative unfamiliarity and perceived complexity. Thus, we are presenting complementary papers to demonstrate issues relating to causal estimation, along with practical recommendations for trial analysts planning to undertake causal estimation.

In the first of these publications,^4^ we describe various causal research questions of interest in a range of clinical trial settings, demonstrating when and why it is important to allow for treatment departures in the analysis of clinical trials. In the present paper, we demonstrate application of the SFTM and IPCW in a trial of chronic disease with longitudinal treatment periods, multiple types of treatment changes (rather than one-way switches alone), and survival (or ‘time to event’) outcomes. We compare the suitability of these methods in this trial scenario, culminating in guidance for analysts on the choice of methods to use in similarly complicated trials with complex treatment changes and survival outcomes.

## 2 Methods

### 2.1 SANAD trial

The SANAD B trial^5^ compared a number of standard and new antiepileptic drugs among patients diagnosed with generalised epilepsy. This disease area features typical complications when estimating treatment efficacy in a chronic disease where changes to prescribed treatment are common.

The SANAD trial had a pragmatic design, permitting changes to randomised treatment (such as changes to prescribed dose and switching to or addition of other treatments). Patients often experienced multiple treatment changes from randomised treatment over the trial follow-up period.

Despite this pragmatic approach to treatment changes, there was intrinsic clinical interest not only in the pragmatic question of treatment effectiveness but also in estimating the efficacy of randomised treatments, factoring out changes in prescribed treatment from that originally randomised. It was particularly important to consider appropriate causal methods to estimate efficacy of treatment in the SANAD trial because of its non-inferiority (NI) design. At the time of the trial, existing standard antiepileptic drugs (AEDs) with proven efficacy had the disadvantage of a poor side-effect profile; thus, if a newer treatment was shown to be more tolerable, it was deemed necessary only to demonstrate NI of the newer treatment with respect to efficacy in terms of seizure control. Statistical analysis to demonstrate NI (or equivalence) is complicated when treatment changes occur, because such changes typically result in merging of treatment experiences across treatment arms, leading to treatment effects between randomised groups that are more similar than would have otherwise been observed. As such, ITT analysis is anticonservative, necessitating estimation methods beyond both ITT and PP, as both are likely to be biased in this context.^6,7^ PP analysis would involve informative censoring at the time of initial treatment failure, thus introducing bias as initial treatment failure is related to prognosis.

### 2.2 Treatments compared

Although SANAD B featured three randomised treatments, greatest clinical interest lies in the comparison between two of these treatments, sodium valproate (VPS, the standard treatment for patients with generalised epilepsy at the time) and lamotrigine (LTG, the newest and most promising alternative drug).

### 2.3 Clinical outcome

Causal methods are presented here for the primary outcome ‘time to 12 month remission’ (T12mR), defined as the time from randomisation to reaching a 12-month period free of seizures. Remission is often of primary importance from the patient’s perspective in longitudinal trials of chronic disease, but introduces complications in analysis as it represents a non-standard summary of repeated events, with remission impossible by definition within the first 12 months but very common at exactly 12 months.

**Figure 1.**
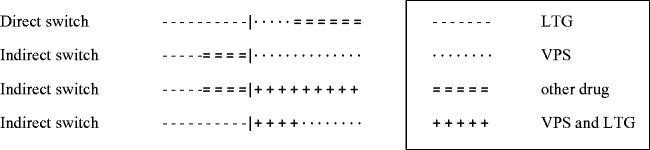
Examples of direct and indirect switches (occurring at the point indicated by ‘|’) for patients randomised to LTG.

### 2.4 Nature of treatment changes

Patients typically experienced multiple forms of treatment changes over the course of the trial, including changes to prescribed dose, switching to a different trial (or non-trial) treatment, or addition of other trial (or non-trial) treatments. We adjust for changes in prescribed treatment, ignoring changes to prescribed dose (as treatment dose is not specified within the protocol, and dose titrations are necessary for loading and withdrawal phases) and patient adherence to their prescriptions (as adherence data were not available). Changes in prescribed treatment are defined as follows: *initial treatment failure* is defined as each patient’s first treatment change of any type following randomisation (excluding changes to prescribed dose of randomised treatment); a *switch* is defined as starting the rival trial treatment, of which a *direct switch* is defined as stopping randomised treatment and starting the rival trial treatment (i.e. switching from VPR to LTG or vice versa) at the point of initial treatment failure (i.e. the first of any treatment changes) and an *indirect switch* is any switch to the rival trial treatment after the point of initial treatment failure (or adding the rival trial treatment at any time) (see figure 1).

The reason for treatment change is available only at the point of initial treatment failure and is classified either as inefficacy of treatment (manifested by inadequate seizure control, ISC), intolerability (unacceptable adverse effects, UAEs) or patient choice.

### 2.5 Estimands: Explanatory versus pragmatic causal research questions

The question of interest may reflect a purely explanatory clinical question (‘what are the relative effects of LTG versus VPS?’), thus comparing only the effects of the two treatments of interest and excluding the effect of any other treatment received during the trial treatment period. For this estimand we would need to factor out (i.e. remove the effect of) all treatment changes, using methods that will be described below.

However, clinicians recognise that in practice, after a decision is made to change from the original randomised treatment, patients may first try other treatments or combinations before ending up on the alternative treatment of interest. Thus, a more realistic and pragmatic causal analysis allows that patients may be offered treatments and try them for a short period before deciding whether to take them long term and assumes that these short exposures to other treatments do not overly influence the effect on sustained freedom from seizures. This ‘pragmatic-causal’ question asks

what are the relative effects of LTG (with any treatment changes that ensue with this treatment policy, excluding adding or switching to VPS) versus VPS (with any treatment changes that ensue with this treatment policy, excluding adding or switching to LTG)?

For this estimand, we would need to factor out switches and additions to the alternative treatment of interest (VPS or LTG) but no other treatment changes.

Treatment changes prior to T12mR due to UAEs are inevitable and necessary, but clinicians argue that changes due to ISC may be avoided by more appropriate dosing of randomised drug. It was therefore of particular interest to supplement ITT analysis with a causal analysis adjusting only for treatment changes due to ISC, in order to assess the relative benefit of each drug in achieving a minimum T12mR in the absence of any changes to prescribed treatment due to ISC (i.e. as if the clinician had appropriately adjusted the prescribed dose to control seizures).

As the reason for treatment change was collected only for the first treatment change per patient (at the point of initial treatment failure), it was not possible to consider the impact of specific reasons for indirect switches for the pragmatic-causal estimand (as these were mostly not the first treatment change for a given patient but instead occurred subsequent to another form of treatment change). Estimands relating to reason for treatment change were therefore only estimable when factoring out treatment changes at the point of initial treatment failure. This was implemented by performing a sequence of estimands, accounting for reasons for initial treatment failure in the order determined according to clinical importance. As clinicians were primarily interested in factoring out initial treatment failure due to ISC, this was the first estimand in the sequence. The second estimand accounted for initial treatment failure due to ISC or personal choice (e.g. non-compliance or potential to become pregnant), and the final estimand accounted for all initial treatment failure (including those due to UAEs also, which is considered the least easily altered treatment change). The estimands are listed in Table 1.

**Table 1.** Estimands and analyses.

	Estimand	Possible analyses		Analysis chosen
		SFTM	IPCW	
Pragmatic estimands				
ITT (no treatment changes)	Observed treatment effect demonstrating effectiveness of treatment assignment	✓	✓	ITT
Pragmatic-causal estimands				
All switches (due to ISC, choice or UAEs)^a^ (Sw3)	Treatment effect estimate that would have been observed if it had been possible to prevent (i.e. factor out) all treatment switches (direct and indirect)	✓	✓	SFTM^b^
Explanatory estimands				
Direct switches due to ISC alone (Dir1)	Treatment effect estimate that would have been observed if it had been possible to prevent (i.e. factor out) direct treatment switches occurring due to ISC (of primary interest to clinicians)	✓	✓	SFTM^b^
Direct switches due to ISC or choice (Dir2)	Treatment effect estimate that would have been observed if it had been possible to prevent (i.e. factor out) direct treatment switches occurring due to ISC or for reasons of personal choice	✓	✓	SFTM^b^
All direct switches (due to ISC, choice or UAEs) (Dir3)	Treatment effect estimate that would have been observed if it had been possible to prevent (i.e. factor out) all direct treatment switches	✓	✓	SFTM^b^
All initial treatment failures due to ISC alone (TF1)	Treatment effect estimate that would have been observed if it had been possible to prevent (i.e. factor out) initial treatment failure occurring due to ISC (of primary interest to clinicians)		✓	IPCW
All initial treatment failures due to ISC or choice (TF2)	Treatment effect estimate that would have been observed if it had been possible to prevent (i.e. factor out) initial treatment failure occurring due to ISC or for reasons of personal choice		✓	IPCW
All initial treatment failures (due to ISC, choice or UAEs) (TF3)	Treatment effect estimate that would have been observed if it had been possible to prevent (i.e. factor out) all initial treatment failures		✓	IPCW/PP

PP: per protocol; IPCW: inverse probability of censoring weighting; SFTM: structural failure time model; ITT: intention to treat; UAEs: unacceptable adverse effects; ISC: inadequate seizure control.

a It was not possible to consider the breakdown according to reason for treatment change for indirect switches, as the reason for treatment change was recorded for the first treatment change only; by indirect switches mostly occurred after the initial treatment change, in which case the reason for treatment change was not available.

b SFTM was chosen in preference to IPCW, in order to avoid the NUC assumption where possible.

### 2.6 Simple estimation methods

Data were initially analysed with ITT (adjusting for no switches and estimating a fully pragmatic estimand) and PP (censoring at the time of each patient’s first treatment change and thus estimating the explanatory estimand TF3).

### 2.7 Causal estimation methods

#### 2.7.1 SFTM

The SFTM is based on the potential outcomes framework, under which each randomised individual has an underlying (‘counterfactual’) outcome that would have been observed if they had received control treatment (VPS). Estimation of the causal treatment effect is based on the assumed balance between randomised arms in these underlying outcomes which we define as $U_{0i}$ for the *i*th individual. If the data included direct switches only, we could divide follow-up $T_{i}=T_{0i}+T_{1i}$ where $T_{0i}$ is time spent on control treatment and $T_{1i}$ is time spent on experimental treatment. Then, we could fit an accelerated failure time model:

$$U_{0i}=T_{0i}+e^{\beta }T_{1i}$$

where $e^{\beta }$ reflects the expansion (or contraction) in survival time attributable to experimental treatment and is called the acceleration factor (AF).^8^ The AF is interpreted in terms of rate of ‘using up’ survival time; in this example, an AF < 1 implies that T12mR is achieved more slowly with LTG compared to VPS, and AF > 1 implies that T12mR is achieved more quickly with LTG. This model can then be applied to generate each patient’s survival time that would have been observed if no treatment switches had occurred. These counterfactual data can then be used to estimate the ‘corrected’ hazard ratio (HR), if it can be assumed that the survival times follow a Weibull distribution $h(t)=\varphi \gamma t^{\gamma -1}$ (where φ denotes the scale parameter and γ the shape parameter),^9^ as this model provides direct correspondence between the accelerated lifetime model and Cox model (such that the RPSFTM estimand β can be expressed in terms of the Cox parameter θ, as β=θ/θγγ). If one can assume the exponential distribution (γ=1), the HR ($e^{\theta }$) and AF ($e^{\beta }$) will therefore be equal.

To employ this method in SANAD, it is necessary to define ‘control’ and ‘experimental’ treatment times ($T_{0i}$ and $T_{1i}$) relating to the particular causal question of interest.

For the pragmatic-causal estimand, adjusting for all (indirect and direct) switches, $T_{0i}$ is defined as the total time spent on a VPS-based treatment policy, including time following any other additions or switches to subsequent treatments, but excluding addition with, or switching to, LTG. Similarly, $T_{1i}$ is defined as the total time spent on an LTG-based treatment policy, including any other additions or switches to subsequent treatments, but excluding addition with, or switching to, VPS. With these definitions, a switch from VPS to LTG may be regarded as starting an LTG-based treatment policy, so that time before a switch is included in $T_{0i}$ and time after a switch is included in $T_{1i}$ (and similarly for a switch from LTG to VPS). Time spent with the rival treatment prescribed in addition to the randomised treatment does not fit well into this framework; we included it within $T_{1i}$ if the patient was randomised to VPS and within $T_{0i}$ if the randomised treatment was LTG (i.e. addition of the rival treatment is considered as time spent on the rival treatment). In evaluating the effect of ‘ending up on’ the alternative treatment, this analysis considers any other changes to treatment as a pragmatic continuation of randomised (or switched) treatment, thus removing the need to censor follow-up at the point of these alternative treatment changes.

For the explanatory estimand, we have no alternative but to censor individuals whose first treatment change is not a direct switch, and thus risk selection bias. We then define $T_{0i}$ as time spent on VPS and $T_{1i}$ as time spent on LTG. As this analysis factors out direct switches only, it was possible to estimate separate explanatory estimands accounting for the reasons for these switches (ISC, personal choice or UAEs) as described in Table 1.

##### 2.7.1.1 Recensoring and G-estimation

A major difficulty with the RPSFTM is that non-informative censoring (on the original T-scale) may become informative on the U-scale through its inherent association with treatment received. Robins and Tsiatis^10^ proposed the use of recensoring to remove this dependence of censoring time on treatment history, in order to allow unbiased estimation of the causal parameter. This is achieved by recensoring each individual’s survival time at the minimum of all possible censoring times over all possible treatment history patterns in their allocated treatment group. This method requires that a potential censoring time on the original T-scale is provided for each individual (for example, as a fixed maximum follow-up time for all individuals or a maximum follow-up time for each individual based on the difference between their date of entry and the final date of follow-up).

As the SFTM is reliant on unobserved ‘counterfactuals’, the acceleration factor cannot be estimated using usual methods for associational models. Instead, a method known as *G-estimation* is employed based on the assumption that the underlying control treatment survival times $U_{0i}$ can be considered a baseline feature unaffected by post-randomisation treatment or confounding, which on average will differ only randomly between the randomised groups. G-estimation is based on finding a value of the treatment effect parameter (β) that attains baseline balance in terms of potential outcome $U_{0i}$ between randomised groups.^11^

Another key implicit assumption of the SFTM is the ‘constant treatment effect’, i.e. that the effect of treatment does not vary according when it is taken relative to the state of the patient or disease progression.

### 2.8 SFTM implementation

The SFTM was run using the strbee code in Stata.^12^ Technical details and Stata code for the SFTM procedure can be found in the supplementary material.

### 2.9 IPCW

In the context of a randomised controlled trial with a survival outcome, the IPCW method can be used to adjust for changes from randomised treatment by artificially censoring patients at the point of the first of any (relevant) treatment change. The potential bias introduced by this censoring is addressed by weighting the remaining (uncensored) patients by the inverse of their probability of remaining uncensored. These probabilities are determined conditional on all factors that jointly predict outcome and treatment change, thus removing dependence between outcome and censoring under the assumption of no unmeasured confounders (NUC). The reasoning behind this methodology is that, if one is able to determine all factors that jointly predict treatment change and outcome, weighting up the analysis of time to outcome (by the inverse probability of remaining uncensored) while censoring at the point of any treatment change means that the censored outcomes of individuals who *have* changed treatment are unbiasedly represented by the up-weighted outcomes (WOs) of ‘similar’ individuals who have *not* changed treatment. Thus, the assumption of NUC is key, as is that of *positivity* (that the probability of remaining on treatment is above zero for all possible covariate combinations at each time point) in order to ensure weights (inverse of these probabilities) are estimable throughout follow-up.^13^

There are three main steps when applying IPCW to randomised trial data with time-varying covariates (TVCs), as in SANAD. Firstly, it is necessary to determine which (baseline and time-varying) covariates influence the probability of switching and outcome. Secondly, these covariates are included in a weight determining (WD) model (either a Cox model for time to (first) treatment change or pooled logistic regression (PLR) for discretised interval data with treatment change as the dependent variable) in order to generate time-varying stabilised weights for each individual. Thirdly, these stabilised weights are applied in the WO model, regressing time to event on randomised treatment group and baseline covariates only and artificially censoring patients when they deviate from their assigned treatment.

Further information on the considerations necessary for the IPCW analysis for this trial can be found in the supplementary material.^28–30^

### 2.10. IPCW implementation

#### 2.10.1. Discrete or continuous time

The IPCW method can be applied in continuous time, in which case the WD and WO models are fitted using Cox regression and reported using hazard ratios (HR). Alternatively, it can be applied in discrete time, in which case the WD and WO models are fitted using PLR and reported using odds ratios (OR).

##### 2.10.1.1.Width of time interval

It was necessary to decide on the optimal length of time interval for the PLR discretised outcomes and TVCs (for both the PLR and Cox models, as explained below), in order to strike the balance between greater accuracy (which increases as interval length decreases) and computational intensity (which decreases with interval length). Taking into account the frequency and duration of follow-up information in this analysis (median [range] of follow-up = 1 year [2 weeks, 5 years] with the potential for covariate information to be updated on a daily basis), it seemed sensible to use fortnightly intervals to define the PLR outcome and TVCs, with sensitivity analyses using weekly and monthly intervals.

#### 2.10.2 Selection of variables in WD model

Expert clinical opinion was sought to determine the key determinants of treatment change and outcome. Experts expected a number of TVCs to impact on probability of censoring, but were unsure whether baseline variables would have an independent effect beyond these TVCs. As such, a variable selection process was used to determine which of the many baseline and TVCs should be adjusted for when applying this IPCW model to the SANAD data, to avoid the possibility of overfitting the model (leading to model instability due to a low event per variable, EPV, ratio). The CHEST (CHange in ESTimate) criterion is preferable to using p-values to determine which variables should be included;^14^ this method assesses the change in treatment effect (HR or OR) when each potentially confounding variable is included (if using forward selection, FS) or excluded (if using backward elimination, BE) from the IPCW model, using a relatively small threshold (suggested maximum of 10%) to determine whether each variable should be included. For example, when using BE, if exclusion of the variable (from the WD model) causes the treatment effect HR (in the WO model) to change by more than the chosen threshold (e.g. 1%), the variable would be retained.

Practical considerations for this selection process included the choice of threshold for inclusion of covariates, choice of whether to use forward or backward selection procedures and the inherent reliability of the selection procedure in terms of EPV ratios.

##### 2.10.2.1. TVCs

Suitable TVCs are variables which affect the probability of both treatment change and remission. The first obvious TVC was cumulative seizure count since randomisation. Given that some patients experienced multiple daily seizures, it was convenient to create cumulative interval-based counts of seizures, leading to a step function that changed on an interval (for example, monthly or weekly) basis. Two other obvious TVCs were the occurrence of AEs (weighted up by the number of nights in hospital) and dose of randomised treatment (recorded at each clinic visit and assumed to be unchanged and adhered to between visits). All TVCs were fixed at their value at the start of the interval, such that this value was then assumed to stay constant for that entire time period (to ensure that the values were not affected by any event occurring within that interval). For example, the cumulative AE (or seizure) counts for time interval *n* equalled the total up to the end of interval *n*−1. Similarly, the interval value for dose at a particular interval was the last recorded dose from within the previous interval. These interval values were used for both the PLR and Cox models.

##### 2.10.2.2. Covariate issues relating to stability of model

In order to reduce the potential impact of the highly skewed distribution of all of the continuous baseline and TVCs on the stability of the model, these variables were truncated at their 1% and 99% centiles (as recommended by Royston and Sauerbrei^15^ to prevent unstable modelling due to overly influential extreme values).

##### 2.10.2.3. Extreme weights

Very large values of weights are undesirable because they reduce the effective sample size. A potential cause of extreme weights is an incorrect specification of the functional form for covariates in the PLR model, thus implying an incorrect relationship between each covariate and the outcome. In particular, if the model wrongly assumes linearity (i.e. when untransformed covariates are included in the model), patients with extreme values of TVCs become disproportionately influential, in which case alternative models with log or inverse links (that asymptote rather than increase linearly as the TVC increases) should be explored.

Martingale residuals are useful in determining the most appropriate functional form of covariates to be included in the model. A locally weighted smoothing (lowess) curve of the martingale residuals from the (constant only) Cox model plotted against each transformation of each TVC in turn (untransformed values (X), logged values (log(1 + X)) and inverse values (1/(1 + X))) will suggest an appropriate transformation. On visual inspection of these smoothing curves, it became apparent that the logarithmic transformation was appropriate for the TVC seizure count and three baseline continuous variables (interval between first ever seizure and randomisation, total number of tonic clonic seizures prior to randomisation, age).

#### 2.10.3. Controlling for time in PLR

When using PLR, a spline function of time can be used to mimic the underlying hazard function of Cox regression, thus allowing the underlying risk of the event to vary from interval to interval while avoiding the need for a separate intercept term for each interval.^16^ If the hazard is likely to change shape or be particularly changeable at a certain time, one or more knots should be placed near these change points.^16^ With a rare outcome, it is advantageous to fix the knot positions at centiles of the distribution of *observed* event times, rather than all observation times.

Different spline variables must be created for the WD and WO models; these should be treatment-specific for the WD model, with knots placed at centiles of observed treatment change times in each treatment group separately, whereas the WO spline variables are positioned according to centiles of the observed outcome times for the overall trial.

When choosing the number of knots to be used when fitting a spline, one should aim for balance between allowing sufficient flexibility without overfitting the model to the data, which leads to loss of precision.^17^ The chosen number of knots impacts on the EPV ratio, as a spline with *k* knots will require *k*−1 parameters, in addition to the coefficient for the linear time variable (which necessarily accompanies the spline variable), thus adding *k* parameters in all.

When considering the positioning of knots for the WO model, in the particular case of SANAD, it was important to recognise that the remission outcome could not (by definition) occur prior to 12 months. However, at 12 months, there was then a peak in the number of events occurring, due to a high proportion (approximately 30%) of patients achieving immediate 12-month remission (I12mR). This major non-linearity (spike) of the underlying hazard at 12 months presented a challenge for the spline variable to adequately fit the data. We found that this pattern was best fitted by dropping any time at which no events occurred (thus excluding times less than 12 months) and fitting a binary indicator variable at 12 months (to capture the peak in remission events at this time point) and a linear time variable beyond that point.

Spline variables were created in Stata using the command spbase.

### 2.11. Method comparison

An analysis plan was developed to facilitate a structured approach to investigate the relative impact on model results of each of the variable factors in this clinical scenario and ultimately to compare the four clinical scenarios of interest (according to reasons for treatment changes). There were four factors to consider: model type (Cox regression or PLR), time intervals for discretised TVCs (weekly, fortnightly or monthly), selection procedure (FS or BE) and variable selection threshold (2%, 5% or 10%). In order to reduce the likelihood of overfitting, variables were included in the initial pool of baseline and TVCs only if their inclusion in the treatment-only WD model altered the WO treatment effect by at least 1%. Patient follow-up was censored if they had missing seizure count data. Bootstrapping was carried out by drawing 200 repeat samples at the patient level (in other words, selecting the entire record for that patient) to correctly account for clustering.

It was necessary to fit a standard set of models adjusting for the same covariates for each of the model type/treatment change/time interval combinations, in order to allow direct comparisons between each analysis scenario. The variable selection procedures demonstrated that baseline covariates were not usually selected into the WD model, but TVCs were often selected, most commonly seizures but also dose and occasionally AE counts. Thus, for the standardised analysis, IPCW analyses were conducted using Cox and PLR models, adjusting for an increasing number of TVCs (none; seizures alone; seizures and dose; seizures, dose and AEs) for each set of treatment changes (none; treatment changes due to ISC alone; treatment changes due to ISC or personal choice; all treatment changes due to ISC, personal choice or UAEs) and each time interval (month; fortnight; week). Stabilised weights were created by multiplying the inverse of the probability of censoring (obtained from the WD model adjusted for TVCs) by the probability obtained from the empty WD model (as there were no baseline covariates included in this analysis), separately for each treatment arm. Stata code for the PLR and Cox IPCW models is provided in the supplementary appendix.

## 3 Results

A total of 477 patients were randomised to LTG (239) and VPS (238); for ease of exposition, we analyse the 387 (81%) who had complete data on all baseline and TVCs. The number (%) of these patients who underwent any treatment changes or switches is displayed in Table 2, along with the reason for direct switches and (the first of) any treatment changes. The reason for indirect switches was not always available, as these switches were not necessarily the first treatment change for each patient. Direct switches were twice as common (and switches generally were nearly twice as common) in LTG compared with VPS patients, but only 59% (and 53%) of the total number of switches that occurred in the LTG (VPS) arm were eligible to be analysed as direct switches.

**Table 2.** Frequency of treatment changes and switches between VPS and LTG.

Treatment changes	LTG (n = 193)	VPS (n = 194)	Total (n = 387)
Initial treatment failure (first of any treatment change)	74 (37%)	59 (30%)	133 (34%)
Treatment changes due to ISC alone (TF1)	52	26	
Treatment changes due to ISC or personal choice (TF2)	55	29	
All treatment changes (due to ISC, personal choice or UAEs) (TF3)	74	59	
Switches (between LTG and VPS) (Sw3)	59 (30%)	34 (17%)	93 (24%)
Indirect switch	24 (12%)	16 (8%)	40 (10%)
Direct switch	35 (18%)	18 (9%)	53 (13%)
Direct switches due to ISC alone (Dir1)	25	9	
Direct switches due to ISC or personal choice (Dir2)	25	11	
All direct switches (due to ISC, personal choice or UAEs) (Dir3)	35	18	

UAE: unacceptable adverse effect; ISC: inadequate seizure control; LTG: lamotrigine; VPS: sodium valproate.

Results for the SFTM and IPCW models are presented in Table 3. As expected, artificial censoring in PP analysis (at the time of each patient’s initial treatment change) and the direct switches SFTM (at the time of all initial treatment changes other than direct switches) curtail analysis times, as demonstrated by the drop in median (interquartile range, IQR) survival times in the PP analysis and to a lesser degree in the direct switches SFTM compared to the ITT and all switches SFTM analysis, neither of which introduce artificial censoring.

**Table 3.** SFTM and IPCW results (with fortnightly intervals) for LTG:VPS.

Estimand	Follow-up time:median (IQR) days		Number (%) with remission		Number (%) with treatment change		IPCW		
	LTG (n = 193)	VPS (n = 194)	LTG (n = 193)	VPS (n = 194)	LTG (n = 193)	VPS (n = 194)	TVCs included in WD model	PLR OR (95% CI)	Cox HR (95% CI)
ITT	385 (365, 646)	517 (365, 876)	143 (74%)	154 (79%)			(No TVCs included in model)	0.72 (0.57, 0.94)	
							(*Breslow*)		0.77 (0.61, 0.97)
							(*Efron*)		0.73 (0.58, 0.92)
							(*Exact partial likelihood*)		0.72 (0.56, 0.93)
TF3 (PP)	365 (280, 531)	365 (365, 483)	90 (47%)	114 (59%)			(No TVCs included in model)	0.65 (0.48, 0.87)	
							*Breslow*		0.73 (0.58, 0.89)
							*Efron*		0.68 (0.52, 0.90)
							(*Exact partial likelihood*)		0.65 (0.47, 0.90)
TF1	365 (280, 531)	365 (365, 483)	110 (57%)	141 (73%)	52 (27%)	26 (13%)	No TVCs	0.70 (0.55, 0.996)	
							(*Breslow*)		0.77 (0.64, 1.00)
							(*Efron*)		0.73 (0.57, 0.94)
							(*Exact partial likelihood*)		0.70 (0.53, 0.94)
							Seizures	0.68 (0.38, 1.26)	0.74 (0.42, 3.03)
							Seizures and dose	0.67 (0.37, 1.36)	0.73 (0.22, 68.63)
							Seizures, dose and AEs	0.66 (0.39, 12.09)	0.72 (0.29, 120.24)
TF2	365 (280, 531)	365 (365, 483)	108 (56%)	139 (72%)	55 (28%)	29 (15%)	No TVCs	0.70 (0.54, 0.99)	
							(*Breslow*)		0.77 (0.60, 0.99)
							(*Efron*)		0.73 (0.57, 0.94)
							(*Exact partial likelihood*)		0.70 (0.52, 0.94)
							Seizures	0.68 (0.38, 1.35)	0.75 (0.37, 3.11)
							Seizures and dose	0.67 (0.34, 3.38)	0.73 (0.19, 29.71)
							Seizures, dose and AEs	0.66 (0.38, 2.97)	0.72 (0.21, 102.47)
TF3	365 (280, 531)	365 (365, 483)	90 (47%)	114 (59%)	74 (38%)	59 (30%)	No TVCs	0.65 (0.48, 0.87)	
							(*Breslow*)		0.73 (0.58, 0.89)
							(*Efron*)		0.68 (0.52, 0.90)
							(*Exact partial likelihood*)		0.65 (0.47, 0.90)
							Seizures	0.61 (0.40, 1.10)	0.68 (0.53, 1.18)
							Seizures and dose	0.60 (0.40, 1.84)	0.67 (0.47, 2.99)
							Seizures, dose and AEs	0.59 (0.28, 1.54)	0.65 (0.26, 2.58)
								SFTM	
								AF = $e^{\underline{\beta }}$ (95%CI)	HR (95% CI)
Sw3	438 (365, 906)	570 (365, 1162)	143 (74%)	154 (79%)	59 (31%)	34 (18%)		0.77 (0.52, 0.999)	0.76 (0.57, 1.03)
Dir1	379 (365, 684)	365 (365, 485)	110 (57%)	119 (61%)	25 (13%)	9 (5%)		0.89 (0.72, 1.000)	0.73 (0.54, 0.98)
Dir2	379 (365, 684)	365 (365, 485)	111 (57%)	121 (62%)	25 (13%)	11 (6%)		0.89 (0.72, 1.000)	0.72 (0.54, 0.97)
Dir3	394 (365, 692)	365 (365, 496)	116 (60%)	129 (66%)	35 (18%)	18 (9%)		0.84 (0.67, 0.999)	0.68 (0.51, 0.90)

IPCW: inverse probability of censoring weighting; IQR: interquartile range; LTG: lamotrigine; VPS: sodium valproate; PLR: pooled logistic regression; OR: odds ratio; HR: hazard ratio; TVC: time-varying covariate; ITT: intention to treat; CI: confidence interval; PP: per protocol; AE: adverse events; SFTM: structural failure time model.

### 3.1 SFTM

Adjustment for increasing numbers of direct switches between LTG and VPS (from Dir1 to Dir2 to Dir3) causes the estimated AF (and HR) to fall further away from one, with intermediate results between ITT and PP analyses for these three direct switch scenarios (see Table 3). In this example, an AF < 1 implies that T12mR is achieved more slowly with LTG compared to VPS; conversely, AF > 1 implies that T12mR is achieved more quickly with LTG compared to VPS. The SFTM results therefore suggest that T12mR is ‘used up’ increasingly slowly (with LTG compared to VPS) as more direct switches are accounted for (i.e. the benefit of VPS becomes increasingly apparent).

In contrast, the HR obtained when adjusting for all (direct and indirect) switches between LTG and PVS (Sw3) is closer to 1 than the ITT analysis, suggesting a dilution of the ITT treatment effect when accounting for the effects of indirect switches (i.e. those occurring subsequent to initial treatment failure or additions of rival treatment). Note that the AF for the Sw3 analysis is further from 1 than the AFs for the Dir analyses, whereas the HR for the Sw3 analysis is closer to 1 than the HR for the Dir analyses. This apparent anomaly would arise if the baseline hazard functions have different shapes in the Sw3 and Dir analyses, such that the relationship between HR and AF differs between the Sw3 and Dir analyses.

### 3.2 IPCW

IPCW modelling results for PLR and Cox models with fortnightly intervals for each treatment change combination, adjusting for increasing numbers of TVCs, are displayed in Table 3. Generally, as more treatment changes were accounted for, and as the number of TVCs in the model increases, the treatment effect decreased away from 1, suggesting an increasing advantage of VPS. In other words, without departure from randomised assignment, the observed (ITT) treatment effect would have demonstrated a greater advantage of VPS over LTG than that observed in the trial. Time interval width did not seem to influence treatment effects (see supplementary appendix for weekly and monthly results).

Note that confidence intervals (CIs) are asymmetric because they are determined using bootstrapping.

The presence of weights meant it was not possible to account for tied remission times using Efron’s correction or exact partial likelihood in the WO Cox model. This is a potential disadvantage, given that nearly half (143, 48%) of patients who achieved remission did so immediately at 12 months. However, the likely bias when using the standard Breslow method in the presence of ties can be estimated by comparison with the treatment effect obtained from Efron or partial exact likelihood for the corresponding ITT analysis. Once this bias (in the order of 0.07 across all scenarios) is subtracted from the Cox HR treatment effects, there is almost perfect correspondence between treatment effects obtained from the Cox and PLR models.

A lack of convergence was evident in a few bootstrap samples used to determine CIs for PLR ORs, and extreme upper limits were obtained for bootstrapped CIs of Cox HRs. This problem occurred when weight estimation was based on relatively few ‘treatment change’ events (in the ISC or ISC/choice scenarios) while adjusting for a large number of TVCs.

### 3.3 Comparison of IPCW and SFTM

In comparison to the IPCW analyses, adjustment for different reasons for treatment switches using SFTM (Dir1, Dir2 and Dir3) has a less dramatic effect than on the corresponding IPCW treatment effect estimates (TF1, TF2 and TF3). This is as expected given that, in total, treatment switches from LTG to VPS (and vice versa) make up only one half (and one third) of the patient’s first treatment changes accounted for by IPCW. In adjusting only for switches occurring for particular reasons (for example, due to ISC alone), the power of the SFTM is reduced while the bias increases, because of additional necessary censoring when patients experience treatment switches for other reasons.

## 4 Discussion

Despite a pragmatic design, with changes to prescribed treatment permitted due to inefficacy or intolerability of treatment, there was intrinsic interest in the SANAD trial in estimating treatment efficacy in the absence of such treatment switches. However, appropriate causal analysis was complicated by the complex remission outcome and complicated treatment change patterns. We discuss the SFTM and IPCW methods in turn before offering suggestions for choice of method.

### 4.1 SFTM

There were a number of major disadvantages when applying the SFTM in this context.

### 4.2 Censoring

Firstly, the restriction of the SFTM to a single acceleration factor means that only two treatment states could be compared. Thus, in the same way as for the PP analysis sets, all treatment changes other than switches between randomised treatment arms were necessarily censored, even though such censoring is likely to be informative and may therefore introduce selection bias. The pragmatic analysis (adjusting for all direct and indirect switches) requires less artificial censoring than the explanatory (direct switch) analysis but addresses a less clear cut clinical question, distinguishing only generally between randomised (VPS and LTG) treatments and failing to account for the impact of any other trial (or non-trial) treatments.

When interpreting results from the SFTM, it is necessary to consider the suitability of the assumptions of the acceleration factor, namely that there is a constant (over time) and common (over individuals) treatment effect, and that the impact of treatment is immediate and constant, without any carryover effect from previous treatment or inherent effect attributable to (the act of) switching. These assumptions may not hold in the SANAD example; for example, it is known that prescription changes can trigger seizures, and loading (and withdrawal) phases when treatment are introduced (and withdrawn) mean that treatment effects will not be constant over the full treatment period.

### 4.3 Complications of T12mR

The greatest concern regarding the use of the SFTM in the context of the SANAD B trial relates to the complexities introduced by the T12mR outcome. In particular, the SFTM fails to recognise two key features of T12mR. First, by definition, 12-month remission cannot occur before 12 months, representing a discontinuity when modelling the effect of treatment. Secondly, a substantial proportion of patients are expected to achieve an I12mR, resulting in a large peak of events occurring at 12 months. In order to accurately represent these data, the estimated AF must indicate a very high event rate at 12 months with truncation of this treatment effect immediately prior to 12 months, which is not possible given the assumption of constant treatment effect.

Shifting the time axis by 12 months, the STFM could instead be used to adjust the effect of treatment switches on time to *start* of achieving delayed 12-month remission (conditional on not having achieved it by 12 months), estimated in only those patients who did not achieve I12mR. The first 12 months of treatment would need to be accounted for, as this period importantly reflects the short- to medium-term tolerability of AEDs, during which treatment changes are common: for example, three quarters of treatment switches from LTG to VPS (and from VPS to LTG) occurred within the first 365 days. Adjustment using a time-fixed summary measure of compliance until first seizure, or until 12 months if the patient achieved I12mR (for example, the proportion of first 12 months spent on randomised treatment) would introduce bias, given that this post-randomisation summary compliance variable is very likely to be related to prognosis. Instead, a time-varying summary measure of treatment receipt (such as the proportion of the previous 12 months that the patient was on randomised treatment) would be required to capture treatment information from the time of randomisation.

An additional complication would be that those who achieved I12mR (as well as those patients who were censored with less than 12 months of follow-up) would be excluded from the analysis, as their *t*-12 analysis time would be less than or equal to 0; this would result in a considerable loss of information (28% (41%) of LTG (VPS) patients achieved I12mR) and results would provide no information on this important class of patients.

A mixture modelling approach, modelling the probability of I12mR in an appropriate (causal) version of logistic regression, would also not be straightforward, as compliance remains time-dependent despite the simplification to a binary outcome; it would thus not be appropriate, for example, to use the method of Sommer and Zeger,^18^ which instead assumes all-or-nothing compliance. Instead, analysis would need to account for different treatments received within the first 12 months of follow-up (if immediate remission was achieved) or until the time of the first seizure (if a seizure occurred within 12 months of randomisation); treatment information following the first seizure (or 12 months, whichever occurs first) is irrelevant as it has no causal impact on achieving (immediate 12 month) remission. There is no obviously appropriate method to assess a binary outcome (I12mR) with a time-dependent treatment covariate that reflects treatment receipt only up to time of time seizure. For example, the general structural mean model (GSMM) proposed by Vansteelandt^19^ accommodates time-dependent compliance with binary outcomes, but this method assumes no switches from the control to experimental arm, which is invalid in this setting. Thus, mixture modelling did not present a straightforward approach to adjust for treatment changes in SANAD B.

### 4.4 IPCW

There were a number of reasons why the IPCW was likely to perform better than the SFTM in this trial: first, the initial 12-month period being devoid of events will not violate the assumptions of the underlying Cox model used in IPCW, as this model makes use only of the ranking of events, rather than actual event times. Although it was not possible to account for ties when executing the WO model, the likely bias due to using the standard Breslow method can be estimated by comparing the treatment effects with those obtained using the Efron correction or exact partial likelihood for the ITT analysis. Allowance for this likely magnitude of bias lead to very similar results between Cox and PLR models across all modelling scenarios.

The explicit allowance for time-varying confounders (under the NUC assumption) is a particular advantage of the IPCW compared with PP, given that this form of confounding is especially relevant in the SANAD trial, where variables such as prescribed dose and cumulative seizure and AE counts are likely to influence the probability not only of remission but also of treatment changes. For example, a high dose of treatment increases the chance of seizure control while simultaneously increasing the probability of adverse drug reactions, which in turn may trigger treatment changes. Indeed, it may be particularly important to consider adjustment for treatment doses in this trial, given the pragmatic trial design (where prescribed dose was completely at the discretion of clinician, and even the initial dose of randomised drug was not standardised) and lack of blinding.^20^ Accounting for these TVCs demonstrated an increasing advantage of VPS over LTG beyond that observed in the ITT analysis.

The greatest advantage of the IPCW analysis over the SFTM, however, is its ability to deal with any form of treatment change, without the need to bias analysis by exclusion or censoring of patients. Application of the IPCW method to the SANAD data addresses a slightly different research question from that corresponding to the STFM, by estimating treatment effect in the absence of any prescribed changes from randomised AED (rather than just factoring out switches between randomised treatments).

However, when undertaking IPCW modelling, analysts must assess whether the underlying NUC assumption is likely to hold, and whether accurate data are available on all relevant confounders between treatment changes and outcome (for example, information used by clinicians when deciding to change a patient’s treatment prescription or when recommending a treatment switch, as this clinical information will often also relate to outcome). If data on key confounders are missing for certain patients (as they were for 19% of the randomised patients in this example), it is important to compare the distribution of treatment change and outcome times in patients with and without complete data, in order to assess generalisability of analysis based on complete data only and to consider use of imputation methods. Furthermore, it is necessary to determine whether there is a sufficiently large pool of those who did (and did not) change treatment at each particular time point to allow reliable weight estimation; in practical terms, this requires assessment of the proportion changing treatment in relation to the sample size at each time point for each combination of patient factors. Analysts must also be vigilant regarding evidence of extreme weights or coefficients. Bootstrapping for treatment effect CIs in this example suggested potential problems associated with weight estimation when allowing for a number of TVCs based on a relatively small number of censoring events in the WD model, as manifested by a lack of convergence (PLR) or extreme upper CI limits (Cox).

### 4.5 Choice of survival analysis methods

When planning to carry out adjustment for treatment changes in trials with survival outcomes, analysts need to consider which of the available methods would be most appropriate, while recognising that no single method will be appropriate for all circumstances; instead, the performance of each method will depend on the particular trial setting to which it is applied. Latimer et al. have assessed the performance of IPCW and RPSFTM methods in extensive simulation studies.^21–23^ Simulation demonstrated that the performance of these methods depends on whether their underlying assumptions are met (in particular, NUC for IPCW and the common treatment effect for RPSFTM). As the validity of these assumptions will depend on the particular trial scenario, Latimer et al. recommend assessing the suitability of each method on a case-by-case basis and provide an algorithm to aid this assessment.^24^

Tables 4 and 5 provide a reminder for trialists of the modelling assumptions and practical limitations of the alternative methods. Given their differing assumptions and data requirements, neither IPCW nor SFTM methods will be directly applicable in every trial setting. It may be useful to apply both (or alternative variations of these) methods, as a means of assessing the sensitivity of results to the associated assumptions.^25,26^ For example, Latimer et al.^23^ propose the use of two-stage methods when the clinical scenario presents a suitable secondary baseline, when the treatment effect varies according to the timing of treatment (i.e. the common treatment effect is not valid) and when patients tend to switch treatments at a common time point (e.g. at the point of disease progression). If several methods are applied, it is important to provide a discussion of the limitations and potential biases associated with each method when presenting results, as an aid to interpretation.

**Table 4.** SFTM considerations.

Model feature	Consideration required
Common treatment effect	Consider whether treatment effect is likely to be constant regardless of when treatment commenced.
Secondary baseline	If treatment switches tend to occur at a common ‘secondary baseline’ (for example, on progression), at which point treatment effect is likely to differ from treatment started at randomisation (for example, on diagnosis) thus violating common treatment effect, consider instead using two-stage SFTM.^27^
Various forms of treatment change	Consider how to address treatment changes (other than those directly accounted for by definition of ‘on’ and ‘off’ treatment in model) in relation to causal research question of interest: substantial numbers of treatment changes (which are not relevant to the causal scenario in question) will undermine validity of analysis, due to necessary censoring at the time of such changes.
Impact of recensoring	Assess effect of recensoring by checking the number of event times (and events) that were recensored, considering the potential impact on treatment effect estimation (if a treatment-time interaction is possible).
Test model performance	Assess success of G-estimation by comparing the counterfactual distributions (the control state event times estimated by applying the optimal AF to the SFTM) for the treatment and control arms; these should be similar under the randomisation assumption.

SFTM: structural failure time model; AF: acceleration factor.

**Table 5.** IPCW modelling considerations.

Model feature	Consideration required
Selection of TVCs	Consider how best to determine which TVCs are important in predicting treatment change and outcome: consult clinical opinion; may be necessary to apply selection procedure (if numerous TVCs)
Functional form of covariates	Check optimal functional form using lowess curve of martingale residuals (from Cox model)
Extreme covariate values	Truncate at the 99th (or 95th) centile to avoid extreme weights (which in turn distort treatment effect estimate) due to influential outlying values of important predictors of treatment change/outcome
Time intervals (for discretised TVCs)	Strike the balance between greater accuracy (increases as interval length decreases) and computational intensity (increases with interval length)
Model type (Cox or PLR)	PLR is useful if using lagged variables or if TVCs change frequently (and therefore are too complicated to be analysed without discretising)
	Cox modelling avoids the need to consider splines to mirror underlying risk function in PLR model
Splines (for PLR only)	Create and use treatment-specific spline variables for WD model, but use overall splines for the WO model
	Consider shape of underlying risk, in order to identify times where risk changes and thus inform positioning of knots
CI estimation	Estimate CIs using bootstrapping to overcome correlation due to within-patient time-varying weights (in Cox model) and the reduction in SEs due to the weight estimation procedure
EPV ratio	Consider ratio of number of variables in model to number of treatment change events, in particular when considering the number of knots to use for spline variables (spline with k knots requires k variables)
Model assumptions	Consider plausibility of NUC assumption (whether all confounding variables have been accounted for) by seeking clinical expert opinion
	Examine model weights for evidence of violation of positivity assumption: extreme weights may indicate unreliable pool of patients who do (or do not) change treatment at a particular time (within a given subgroup of patients defined by cross-classification of model covariates)

TVC: time-varying covariate; PLR: pooled logistic regression; NUC: unmeasured confounders; WO: weighted outcome; WD: weight determining; IPCW: inverse probability of censoring weighting.

## Supplementary Material

Supplementary material
