# Supplementary material for: Adjustment for treatment changes in epilepsy trials: A comparison of causal methods for time-to-event outcomes
Source: Stat Methods Med Res. 2017 Nov 8;28(3):717–33. doi: 10.1177/0962280217735560 (PMC6419234; doi:10.1177/0962280217735560)
Supplement: Supplementary material [file supplementary_material.pdf]

Supplementary material

IPCW

There are three main steps to fitting the IPCW model: fitting the weight determining model, creating stabilised weights and fitting the weighted outcome model.

Fitting the weight determining model

First, it is necessary to identify the important baseline and time-varying covariates that predict both switching and outcome. If the pool of potential confounders is large, it may be necessary to use some form of variable selection process, to avoid the possibility of overfitting the model (leading to model instability due to a low event per variable (EPV) ratio).

With time-varying covariates (TVCs), it may be helpful to set up panel data, for example if the covariates are updated on a daily basis for some patients such that it would be too complicated to adjust for covariates changing on a daily basis. These panel data are created by partitioning each patient's follow up data into intervals of fixed length, such that TVCs are valid at the start of each interval.

The probability of remaining uncensored (in other words, not experiencing treatment change) at each follow up time point, given these covariates, is then estimated for each patient using the weight determining (WD) model, either a Cox model for time to (first) treatment change or pooled logistic regression (PLR) for the discretised interval data with treatment change as the dependent variable.

It may be more practical to use a discrete time model (based on logistic regression, LR) rather than a model assuming continuous event follow up (survival model) to estimate the probability of remaining uncensored, not only when the TVCs are necessarily discretised into intervals (and therefore naturally lend themselves to discrete time analysis) but also because weight determination analysis with TVCs result in weights that vary within individual over time. Unless the statistical software can allow for within-individual time-varying weights in Cox regression, normal standard errors (SEs) from the Cox model (ignoring the patient

identifier in the analysis) will be inaccurate and it will be necessary to implement bootstrapping for valid confidence interval (CI) estimation.

The incorporation of weights has been shown to reduce SEs (29, 30). Thus bias results when standard methods as used for CI estimation as they regard the weights as fixed, whereas non-standard methods (such as bootstrapping) can explicitly allow for the estimation of the weights, reflecting more accurately the associated reduction in variability. Thus, it may be advantageous to use bootstrapping for CI estimation regardless of the choice (between Cox or PLR) of WD model, in order to appropriately account for the reduction in SE estimates as a result of the weight estimation process.

Regardless of choice of WD model, separate models should be created for each randomised treatment group, as the influence of covariates on the probability of treatment changes are likely to differ according to treatment.

Creating stabilised weights

The WD model is then used to provide time-dependent stabilised weights, which are created for each patient i at each time point k during which they remain under randomised treatment, derived from the inverse of their estimated probabilities (of remaining uncensored). Two separate WD models are in fact required in order to generate these stabilised weights: the **first WD model adjusts for baseline** characteristics only, whereas the **second WD model adjusts for both baseline and time-dependent covariates**. Stabilised weights for each time interval are calculated as the ratio of the cumulative probability of remaining uncensored (due to treatment change) from randomisation until (the end of) that interval given only baseline covariates (in other words, using estimated probabilities from the first WD model) divided by the corresponding probability given both baseline and time-dependent covariates (using estimated probabilities from the second WD model).

Once these weights have been created, it is necessary to check their distribution to identify any extreme weights, which may occur for a number of reasons. Firstly, if the majority of patients in a given subgroup (defined by the cross-classification of the baseline and time-

varying covariates included in the WD model) change treatment, the remaining patients will be assigned very large weights to account for the huge loss of information from all other patients in this subgroup. Extreme weights will also result if an incorrect functional form for covariates is specified in the WD model, as predictions of probabilities are then based on an incorrect relationship between each covariate and the outcome.

Fitting the weighted outcome (WO) model

Once any problems regarding weights have been addressed, the stabilised weights are applied to the WO model of time to outcome event, regressed on **randomised treatment group and baseline covariates only** and artificially censoring patients when they deviate from their assigned treatment (i.e. patients who change treatment are assigned a weight equal to 0 (inducing censoring) in all intervals after treatment change).

As for the WD model, when the probability of the outcome event in each time interval is relatively small, the cumulative probability of remaining uncensored (obtained by multiplying each person's estimated probabilities for each successive month of follow up) estimated using PLR will be approximately equal to the probability obtained using a Cox model (31).

The randomised treatment indicator and baseline covariates included in the WD model are included as covariates in the WO model, but the time-dependent covariates are not included, given that their influence has been removed via the weighting process; indeed adjusting for TVCs in the WO model would introduce bias as would any standard analysis regressing on TVCs.

Supplementary Table 1 Weights for IPCW (with fortnightly intervals)

		Weights: mean (SD) [range]		CV of weights (within each fortnight): mean (SD) [range]	
		PLR	Cox	PLR	Cox
**Estimand**	**TVCs included in model**				
**ITT**	(No TVCs included in model)	-	-		
**TF1**					
	No TVCs	-	-		
	Seizures	1.03 (0.30) [0.61, 4.14]	1.03 (0.32) [0.60, 4.61]	0.38 (0.16) [0.003, 0.64]	0.40 (0.18) [0, 0.70]
	Seizures and dose	1.03 (0.47) [0.55, 9.25]	1.04 (0.57) [0.54, 12.52]	0.50 (0.35) [0.003, 1.34]	0.57 (0.45) [0.003, 1.69]
	Seizures, dose and AEs	1.03 (0.46) [0.55, 8.85]	1.04 (0.55) [0.56, 11.84]	0.49 (0.33) [0.003, 1.27]	0.55 (0.43) [0.003, 1.59]
**TF2**					
	No TVCs	-	-		
	Seizures	1.03 (0.31) [0.62, 4.09]	1.03 (0.32) [0.61, 4.41]	0.38 (0.16) [0.003, 0.63]	0.40 (0.17) [0, 0.68]
	Seizures and dose	1.04 (0.48) [0.55, 9.31]	1.04 (0.57) [0.54, 12.43]	0.51 (0.35) [0.003, 1.34]	0.57 (0.45) [0.003, 1.69]
	Seizures, dose and AEs	1.03 (0.46) [0.55, 8.98]	1.04 (0.55) [0.55, 11.83]	0.50 (0.34) [0.003, 1.29]	0.56 (0.43) [0.003, 1.60]
**TF3**					
	No TVCs	-	-		
	Seizures	1.03 (0.21) [0.68, 2.58]	1.03 (0.26) [0.57, 3.30]	0.25 (0.13) [0.002, 0.49]	0.30 (0.19) [0, 0.65]
	Seizures and dose	1.02 (0.24) [0.61, 2.63]	1.03 (0.28) [0.56, 3.85]	0.27 (0.15) [0.004, 0.53]	0.33 (0.21) [0.003, 0.76]
	Seizures, dose and AEs	1.04 (0.38) [0.59, 5.94]	1.04 (0.48) [0.49, 11.75]	0.42 (0.34) [0.004, 1.19]	0.49 (0.46) [0.004, 1.63]

Supplementary Table 2 IPCW with weekly intervals

WD model		IPCW		Weights: mean (SD) [range]		CV of weights (within week): mean (SD) [range]	
		PLR OR (95% CI)	Cox HR (95% CI)	PLR	Cox	PLR	Cox
**Estimand**	**TVCs included in model**						
**ITT**							
	(No TVCs included in model)						
	Breslow		0.77 (0.61, 0.97)				
	Efron		0.73 (0.58, 0.92)				
	Exact partial likelihood		0.72 (0.56, 0.93)				
**TF1**							
	No TVCs						
	(Breslow)		0.77 (0.62, 0.96)				
	(Efron)		0.73 (0.57, 0.94)				
	(Exact partial likelihood)		0.70 (0.53, 0.94)				
	Seizures	0.68 (0.28,1.74)	0.75 (0.35, $1.7*10^{6}$)	1.04 (0.35) [0.60, 5.04]	1.03 (0.33) [0.59,4.94]	0.42 (0.20) [0, 0.78]	0.42 (0.19) [0, 0.77]
	Seizures and dose	0.68 (0.30,2.80)	0.73(0.14,$3.7*10^{7}$)	1.04 (0.55) [0.55, 11.55]	1.04 (0.62) [0.54,14.09]	0.55 (0.42) [0, 1.59]	0.59 (0.49) [0, 1.85]
	Treatment, seizures, dose and AEs	0.64 (0.38,1.63)	0.71(0.14,7162.69)	1.04 (0.48) [0.55, 8.83]	1.04 (0.50) [0.54, 10.33]	0.48 (0.33) [0, 1.25]	0.52 (0.37) [0, 1.41]
**TF2**							
	No TVCs						
	(Breslow)		0.77 (0.62, 0.96)				
	(Efron)		0.73 (0.57, 0.95)				
	(Exact partial likelihood)		0.70 (0.53, 0.94)				
	Seizures	0.69 (0.27,1.45)	0.75 (0.31, 5.33)	1.04 (0.34) [0.60, 4.76]	1.04 (0.33) [0.60, 4.68]	0.41 (0.19) [0, 0.75]	0.41 (0.18) [0, 0.73]
	Seizures and dose	0.68 (0.30,3.11)	0.73(0.14,25375.19)	1.04 (0.56) [0.54, 11.76]	1.04 (0.62) [0.53,13.90]	0.56 (0.43) [0, 1.62]	0.60 (0.48) [0, 1.84]
	Seizures, dose and AEs	0.65 (0.40,1.98)	0.71 (0.12,$4*10^{6}$)	1.04 (0.48) [0.54, 9.23]	1.04 (0.51) [0.53, 10.61]	0.49 (0.34) [0, 1.30]	0.52 (0.38) [0, 1.45]
**TF3**							
	No TVCs						
	(Breslow)		0.73 (0.55, 0.96)				
	(Efron)		0.69 (0.52, 0.91)				
	(Exact partial likelihood)		0.65 (0.47, 0.90)				
	Seizures	0.60 (0.37, 1.16)	0.67 (0.48, 1.86)	1.03 (0.24) [0.65, 2.90]	1.03(0.27) [0.57, 3.39]	0.29 (0.15) [0.002,0.57]	0.32 (0.19) [0, 0.74]
	Seizures and dose	0.59 (0.39, 1.70)	0.66 (0.45, 52.29)	1.03 (0.26) [0.60, 3.00]	1.02 (0.29) [0.56, 3.48]	0.30 (0.17) [0.003,0.62]	0.34 (0.20) [0.002, 0.78]
	Seizures, dose and AEs	0.57 (0.24, 2.65)	0.63 (0.25, 56.09)	1.05 (0.47) [0.56, 6.96]	1.05 (0.52) [0.48, 11.11]	0.48 (0.38) [0.003,1.43]	0.51 (0.44) [0.002, 1.80]

Supplementary Table 3 IPCW with monthly intervals

WD model		IPCW		Weights: mean (SD) [range]		CV of weights (within month): mean (SD) [range]	
		PLR OR (95% CI)	Cox HR (95% CI)	PLR	Cox	PLR	Cox
**Estimand**	**TVCs included in model**						
**ITT**		0.72 (0.57, 0.95)					
	(No TVCs included in model)						
	Breslow		0.77 (0.62, 0.97)				
	Efron		0.74 (0.59, 0.92)				
	Exact partial likelihood		0.72 (0.56, 0.93)				
**TF1**		0.70 (0.51, 0.99)					
	No TVCs						
	(Breslow)		0.77 (0.60, 0.99)				
	(Efron)		0.74 (0.57, 0.95)				
	(Exact partial likelihood)		0.70 (0.52, 0.94)				
	Seizures	0.67 (0.39, 1.16)	0.75 (0.43, 1.34)	1.03 (0.29) [0.61, 3.97]	1.03 (0.30) [0.61, 4.39]	0.35 (0.16) [0.003,0.63]	0.37 (0.17) [0, 0.68]
	Seizures and dose	0.67 (0.34, 1.95)	0.72 (0.27, 94.65)	1.04 (0.63) [0.54, 13.42]	1.05 (0.77) [0.54, 18.02]	0.57 (0.50) [0.004,1.80]	0.65 (0.62) [0.01, 2.19]
	Seizures, dose and AEs	0.66 (0.40, 1.35)	0.71 (0.15, 90.08)	1.04 (0.60) [0.54, 12.71]	1.05 (0.73) [0.54, 16.78]	0.56 (0.48) [0.004,1.71]	0.63 (0.58) [0.01, 2.06]
**TF2**							
	No TVCs						
	(Breslow)		0.77 (0.60, 0.996)				
	(Efron)		0.74 (0.57, 0.95)				
	(Exact partial likelihood)		0.70 (0.52, 0.94)				
	Seizures	0.67 (0.39, 1.16)	0.75 (0.39, 1.23)	1.03 (0.29) [0.63, 3.98]	1.03 (0.30) [0.62, 4.29]	0.35 (0.16) [0.003,0.63]	0.37 (0.17) [0, 0.66]
	Seizures and dose	0.67 (0.32, 1.99)	0.72 (0.25, 507.01)	1.04 (0.63) [0.54, 13.30]	1.05(0.79) [0.53,18.24]	0.58 (0.50) [0.004,1.80]	0.66 (0.62) [0.01, 2.21]
	Seizures, dose and AEs	0.66 (0.40, 1.35)	0.71 (0.27, 72820.67)	1.04 (0.61) [0.54, 12.69]	1.05(0.75) [0.54,17.06]	0.57 (0.48) [0.004,1.72]	0.65 (0.58) [0.01, 2.09]
**TF3**							
	No TVCs						
	(Breslow)		0.74 (0.56, 0.97)				
	(Efron)		0.69 (0.52, 0.91)				
	(Exact partial likelihood)		0.65 (0.46, 0.90)				
	Seizures	0.61 (0.42, 1.35)	0.68 (0.50, 2.06)	1.03 (0.25) [0.62, 3.04]	1.02 (0.26) [0.57, 3.37]	0.30 (0.16) [0.004,0.60]	0.31 (0.19) [0, 0.68]
	Seizures and dose	0.60 (0.38,1.41)	0.66 (0.31, 2.17)	1.03 (0.37) [0.54, 6.80]	1.03 (0.51) [0.51, 10.99]	0.42 (0.33) [0.006,1.24]	0.52 (0.48) [0.01, 1.74]
	Seizures, dose and AEs	0.58 (0.38,1.66)	0.64 (0.30, 2.91)	1.04 (0.36) [0.52, 5.97]	1.04 (0.48) [0.46, 9.62]	0.43 (0.29) [0.006,1.09]	0.53 (0.41) [0.01, 1.50]

Stata code for IPCW PLR and Cox models

Variables

`id`	unique patient identifier
`t_interval`	sequentially numbered within-patient time interval (eg week, fortnight, month from randomisation until remission/treatment change)
`event_t_t0`	variable indicating whether any treatment changes occurred during this time interval for any patients within the VPS group
`event_t_t1`	variable indicating whether any treatment changes occurred during this time interval for any patients within the LTG group
`trt_change`	variable indicating whether patient underwent treatment change during this time interval
`ln_sez`	cumulative seizure count (logged value)
`rem`	variable indicating whether patient achieved 12 month remission during this time interval (1 for remission; 0 for censoring)
`t_1yr`	variable indicating 12 months follow up (ie 0 for all time intervals except 1 year following randomisation)
`t_ge1yr`	variable indicating at least 12 months follow up (ie 0 for all time intervals prior to 12 months)
`event_rem`	variable indicating whether any patients achieved 12 month remission during this time interval
`trt`	treatment group indicator (0 for VPS, 1 for LTG)

IPCW: Pooled logistic regression

```
* Calculate probability of remaining on randomised treatment without
adjusting for TVCs

* Calculate probabilities separately for each treatment group

xi: logistic trt_change t_interval if event_t_t0==1 & trt==0
predict p_t0_t if e(sample)
replace p_t0_t = 0 if event_t_t0==0 & trt==0
replace p_t0_t = 1 - p_t0_t
sort id t_interval
by id: replace p_t0_t = p_t0_t*p_t0_t[_n-1] if _n>1


xi: logistic trt_change t_interval if event_t_t1==1 & trt==1
predict p_t1_t if e(sample)
replace p_t1_t = 0 if event_t_t1==0 & trt==1
replace p_t1_t = 1 - p_t1_t
sort id t_interval
by id: replace p_t1_t = p_t1_t*p_t1_t[_n-1] if _n>1

* Generate combined probability variable including both randomised groups
gen p_t = p_t0_t if trt == 0
replace p_t = p_t1_t if trt == 1

* Calculate probability of remaining on randomised treatment adjusting for
TVC (cumulative seizure count)

* Calculate probabilities separately for each treatment group

xi: logistic trt_change ln_sez t_interval if event_t_t0==1 & trt==0
```

```
predict p_t0_ts if e(sample)
replace p_t0_ts = 0 if event_t_t0==0 & trt==0
replace p_t0_ts = 1 - p_t0_ts
sort id t_interval
by id: replace p_t0_ts = p_t0_ts*p_t0_ts[_n-1] if _n>1

xi: logistic trt_change ln_sez t_interval if event_t_t1==1 & trt==1
predict p_t1_ts if e(sample)
replace p_t1_ts = 0 if event_t_t1==0 & trt==1
replace p_t1_ts = 1 - p_t1_ts
sort id t_interval
by id: replace p_t1_ts = p_t1_ts*p_t1_ts[_n-1] if _n>1

* Generate combined probability variable including both randomised groups
gen p_ts = p_t0_ts if trt == 0
replace p_ts = p_t1_ts if trt == 1

* Calculate stabilised weights
gen w_ts = p_t/p_ts

* Pooled logistic regression with stabilised weights
xi: logistic rem trt t_1yr t_ge1yr if event_rem==1 [pw=w_ts], cluster(id)
```

IPCW: Cox model

```
* Generate probability of remaining on randomised treatment without
adjusting for TVC

stset t_interval, failure(trt_change) id(id)

* Calculate probabilities separately for each treatment group

stcox if trt==0, efron
predict hr_t0_t if trt==0, hr
predict bh_t0_t if trt==0, basehc
* bh_t0_t is only defined on events, therefore
egen bh_t0_tm = mean(bh_t0_t), by(t_interval trt)

stcox if trt==1, efron
predict hr_t1_t if trt==1, hr
predict bh_t1_t if trt==1, basehc
* bh_t1_t is only defined on events, therefore
egen bh_t1_tm = mean(bh_t1_t), by(t_interval trt)

gen ln_p_hr_t = bh_t0_tm * hr_t0_t if trt==0
replace ln_p_hr_t = bh_t1_tm * hr_t1_t if trt==1
sort id t_interval
replace ln_p_hr_t = 0 if ln_p_hr_t==.
by id: replace ln_p_hr_t = ln_p_hr_t + ln_p_hr_t[_n-1] if _n>1
gen p_hr_t = exp(-ln_p_hr_t)

* Generate probability of remaining uncensored (on randomised treatment)
adjusting for TVC (cumulative seizure count)

* Calculate probabilities separately for each treatment group

stcox ln_sez if trt==0, efron
predict hr_t0_ts if trt==0, hr
predict bh_t0_ts if trt==0, basehc
* bh_t0_ts is only defined on events, therefore
```

```
egen bh_t0_tsm = mean(bh_t0_ts), by(t_interval trt)

stcox ln_sez if trt==1, efron
predict hr_t1_ts if trt==1, hr
predict bh_t1_ts if trt==1, basehc
* bh_t1_ts is only defined on events, therefore
egen bh_t1_tsm = mean(bh_t1_ts), by(t_interval trt)

gen ln_p_hr_ts = bh_t0_tsm * hr_t0_ts if trt==0
replace ln_p_hr_ts = bh_t1_tsm * hr_t1_ts if trt==1
sort id t_interval
replace ln_p_hr_ts = 0 if ln_p_hr_ts==.
by id: replace ln_p_hr_ts = ln_p_hr_ts + ln_p_hr_ts[_n-1] if _n>1
gen p_hr_ts = exp(-ln_p_hr_ts)

gen t_hr_ts = p_hr_t/p_hr_ts

stset t_interval [pw=t_hr_ts], failure(rem) time0(prev_t)
stcox trt

* Bootstrap CI required:
scalar hr_ts_scalar = exp(_b[trt])

* myboot command samples the data and returns the statistic of interest
capture program drop myboot
program define myboot, rclass
      preserve
            bsample, strata(trt) cluster(id)
            stset t_interval, failure(trt_change) id(id)
            stcox if trt==0, efron
            predict hr_t0_t_bs if trt==0, hr
            predict bh_t0_t_bs if trt==0, basehc
            egen bh_t0_tm_bs = mean(bh_t0_t_bs), by(t_interval trt)
            stcox if trt==1, efron
            predict hr_t1_t_bs if trt==1, hr
            predict bh_t1_t_bs if trt==1, basehc
            egen bh_t1_tm_bs = mean(bh_t1_tm_bs), by(t_interval trt)
            gen ln_p_hr_t_bs = bh_t0_tm_bs * hr_t0_t_bs if trt==0
            replace ln_p_hr_t_bs = bh_t1_tm_bs * hr_t1_t_bs if trt==1
            sort id t_interval
            replace ln_p_hr_t_bs = 0 if ln_p_hr_t_bs==.
            by id: replace ln_p_hr_t_bs = ln_p_hr_t_bs+ln_p_hr_t_bs[_n-1]//
                  // if _n>1
            gen p_hr_t_bs = exp(-ln_p_hr_t_bs)
            stcox ln_sez if trt==0, efron
            predict hr_t0_ts_bs if trt==0, hr
            predict bh_t0_ts_bs if trt==0, basehc
            egen bh_t0_tms_bs = mean(bh_t0_tsm_bs), by(t_interval trt)
            stcox ln_sez if trt==1, efron
            predict hr_t1_ts_bs if trt==1, hr
            predict bh_t1_ts_bs if trt==1, basehc
            egen bh_t1_ts_bs = mean(bh_t1_ts_bs), by(t_interval trt)
            gen ln_p_hr_ts_bs = bh_t0_tsm_bs * hr_t0_ts_bs if trt==0
            replace ln_p_hr_ts_bs = bh_t1_tsm_bs * hr_t1_ts_bs if trt==1
            sort id t_interval
            replace ln_p_hr_ts_bs = 0 if ln_p_hr_ts_bs==.
            by id: replace ln_p_hr_ts_bs = ln_p_hr_t_sez_bs + //
                  // ln_p_hr_t_sez_bs[_n-1] if _n>1
            gen p_hr_ts_bs = exp(-ln_p_hr_t_sez_bs)
            gen w_hr_ts_bs = p_hr_t_bs/p_hr_t_sez_bs
            stset t_interval [pw=w_hr_ts_bs], failure(rem) time0(prev_t)
```

```
            stcox trt
            return scalar hr_ts_bs = exp(_b[trt])
      restore
end

* Step 3: Simulate treatment effect
simulate hr_ts_bs = r(hr_ts_bs), reps(200) seed(12345): myboot

* Step 4:
bstat, stat(hr_ts_scalar)
estat bootstrap, all
```
